# Distribution of cells responsive to 5-HT_6_ receptor antagonist-induced hypophagia

**DOI:** 10.1016/j.bbr.2014.02.018

**Published:** 2014-06-01

**Authors:** Alastair S. Garfield, Luke K. Burke, Jill Shaw, Mark L. Evans, Lora K. Heisler

**Affiliations:** a Department of Pharmacology, University of Cambridge, Cambridge CB2 1PD, UK; b Centre for Integrative Physiology, University of Edinburgh, Edinburgh EH8 9XD, UK; c Rowett Institute of Nutrition and Health, University of Aberdeen, Aberdeen AB21 9SB, UK; d University of Cambridge Metabolic Research Laboratories, Wellcome Trust-MRC Institute of Metabolic Science, Cambridge CB2 0QQ, UK

**Keywords:** htr6, 5-HT_6_ receptor, SB-399885, Food intake, Hypothalamus, Nucleus of the solitary tract

## Abstract

The central 5-hydroxytryptamine (5-HT; serotonin) system is well established as an important regulator of appetite and continues to remain a focus of obesity research. While much emphasis has focussed on the 5-HT_2C_ receptor (5-HT_2C_R) in 5-HT's anorectic effect, pharmacological manipulation of the 5-HT_6_ receptor (5-HT_6_R) also reduces appetite and body weight and may be amenable to obesity treatment. However, the neurological circuits that underlie 5-HT_6_R-induced hypophagia remain to be identified. Using *c-fos* immunoreactivity (FOS-IR) as a marker of neuronal activation, here we mapped the neuroanatomical targets activated by an anorectic dose of the 5-HT_6_R antagonist SB-399885 throughout the brain. Furthermore, we quantified SB-399855 activated cells within brain appetitive nuclei, the hypothalamus, dorsal raphe nucleus (DRN) and nucleus of the solitary tract (NTS). Our results reveal that 5-HT_6_R antagonist-induced hypophagia is associated with significantly increased neuronal activation in two nuclei with an established role in the central control of appetite, the paraventricular nucleus of the hypothalamus (PVH) and the NTS. In contrast, no changes in FOS-IR were observed between treatment groups within other hypothalamic nuclei or DRN. The data presented here provide a first insight into the neural circuitry underlying 5-HT_6_R antagonist-induced appetite suppression and highlight the PVH and NTS in the coordination of 5-HT_6_R hypophagia.

The physiological regulation of body weight is defined by the homeostatic balance between energy consumption (as calories) and energy expenditure (as basal metabolism, thermogenesis and physical activity). A prolonged surfeit of nutritional energy results in the accumulation of calorific excess as fat, and can ultimately lead to obesity [1,2]. The control of ingestive behaviour is mediated by the central integration of numerous peripherally derived appetitive cues, and the subsequent modulation of neuronal circuits that define the appropriate physiological/behavioural output [2]. In this regard, the central 5-hydroxytryptamine (5-HT, serotonin) system has a well-established function as an anorectic neurotransmitter and is an important neurological determinant of appetite and body weight [3]. Specifically, an increase in 5-HT bioavailability or targeted receptor activation leads to a suppression of food consumption and weight loss [3,4]. Moreover, pharmacological compounds augmenting wholesale 5-HT bioavailability remain amongst the most effective obesity treatments [3,5]. However, off-target effects arising from this generic elevation in 5-HT concentration have prompted more targeted investigation of the specific receptors through which this anorectic action is achieved [3]. Like the G_q_-coupled 5-HT_2C_ (5-HT_2C_R) and G_i_-coupled 5-HT_1B_ (5-HT_1B_R) receptors, recent studies demonstrate that pharmacological manipulation of G_s_-coupled 5-HT_6_ receptor (5-HT_6_R) signalling also suppresses feeding and body weight in rodents, in a manner consistent with the advancement of satiety [6–10]. Unlike the 5-HT_2C_R and 5-HT_1B_R, it is the antagonism rather than the activation of the 5-HT_6_R that is associated with its anorectic function. Consistent with this pharmacological evidence, RNAi mediated central knockdown of 5-HT_6_ expression engenders hypophagia and weight loss, in addition to enhancing Morris water maze performance [8]. Genetic inactivation of 5-HT_6_R signalling, whilst not directly impacting upon basal energy balance, results in resistance to the obesogenic effects of a high-fat diet [11]. Thus, both genetic and pharmacological evidence support a role for the 5-HT_6_R in energy balance regulation.

The 5-HT_6_R is exclusively expressed within the brain [12,13]. These receptors are expressed in multiple regions, but of particular interest, are localised in nuclei of relevance to energy balance regulation such as the arcuate nucleus (ARC), the ventromedial nucleus (VMN) and paraventricular nucleus (PVH) of the hypothalamus and the nucleus of the solitary tract (NTS) [12]. Yet the neurological circuits that underlie 5-HT_6_R antagonist-induced anorexia remain to be identified. Here we report the neuroanatomical regions activated by anorectic concentrations of the 5-HT_6_R antagonist SB-399885.

The appetite-suppressing effect of SB-399885 was investigated in the rat in a paradigm of acute feeding behaviour at concentrations that do not influence exploratory behaviour, depression/anxiety or wakefulness [14,15]. Rats were selected for use because site directed mutagenesis studies indicate that the binding pocket where 5-HT_6_R antagonists bind in human and rat is similar [13]. These and other studies suggest that the rat is a good surrogate species to predict the pharmacology of 5-HT_6_R ligands in humans [9,13]. All procedures were carried out in accordance with the UK Home Office regulations (Science Procedures Act, 1986). Male 250–300 g Sprague Dawley rats (Charles River) were singly housed (56 cm × 38 cm × 17 cm cage) in a temperature (21.5–22.5 °C) and light (12 h on: 12 h off) controlled environment with ad libitum access to regular laboratory chow (EUrodent Diet 22% (protein), PMI Nutrition International) and water, unless otherwise stated. Rats were acclimatised to single housing and regularly handled for one week prior to experimentation. SB-399885 hydrochloride (Tocris) was dissolved in sterile saline. On study days, ad libitum fed rats were administered saline, 1 mg/kg SB-399885 or 2 mg/kg SB-399885 i.p. 45 min prior to the onset of the dark cycle and food was removed from the cages (*n* = 7–8). At the onset of the dark cycle, rats were provided with a known weight of standard chow and food consumption monitored over the following 2 h. A statistically significant dose-dependent suppression of food consumption over the course of the study was observed with 1 mg/kg SB-399885 eliciting a 37% and 2 mg/kg SB-399885 yielding a 78% reduction in food intake compared to saline administration (Fig. 1; One way ANOVA, *F*_2,18_ = 9.35, *p* = 0.016). These data confirm previous observations of 5-HT_6_ antagonist hypophagia [8,10] and support a role for the 5-HT_6_R receptor as a pharmacological target for appetite suppression.

We next sought to identify the neuroanatomical targets that contribute to the anorectic action of SB-399885 as means of elucidating the neurocircuitry underlying the function of such drugs. To reduce endogenous satiety signalling, male Sprague Dawley rats were fasted overnight (16 h) before being administered with saline or 2 mg/kg SB-399885 (i.p.) at the onset of the light cycle when rats are typically less active. Furthermore, food was not provided during this time to prevent nonspecific feeding-associated neuronal activation. Two hours later, rats were deeply anaesthetised (pentobarbitone 50 mg/kg, i.p.) and transcardially perfused with phosphate buffered saline, pH 7.4 (PBS) followed by 10% neutral buffered formalin (Sigma). A second group of male Sprague Dawley rats were ad libitum fed, administered with saline or 2 mg/kg SB-399885 (i.p.) at the onset of the dark cycle and 2 h later were deeply anaesthetised (pentobarbitone 50 mg/kg, i.p.) and transcardially perfused with PBS followed by 10% neutral buffered formalin. Brains were extracted, immersion-fixed in the same fixative for a further 4 h and cryoprotected overnight in 20% sucrose at 4 °C. Brains were cut in coronal section at 25 μm using a freezing microtome and collected as free-floating sections in 6 equal series. One full series of tissue per animal was processed for immunohistochemical detection of the immediate early gene *c-fos* (a molecular marker of neuronal activation) as previously described [16]. Briefly, tissue was washed in PBS and endogenous peroxidases quenched by a 30 min wash in 0.3% H_2_O_2_. After rinsing in PBS, sections were blocked for 60 min in 1% bovine serum albumin (BSA) in PBS/0.1% Triton-X 100 and then incubated overnight at RT in blocking solution containing anti-rabbit cFOS antibody (1/8000; Calbiochem). Sections were then washed and incubated in blocking solution containing biotinylated donkey anti-rabbit IgG secondary antibody (1/1000; Vector Laboratories) for 60 min at room temperature. Following this, sections were incubated in an avidin-peroxidase complex (ABC, Vector Elite kit; 1:250, Vector Laboratories) for 1 h in PBS. The immunoperoxidase was developed using a 3,3-diaminobenzidine tetrahydrochloride kit, as per manufacturer's instructions (Vector Laboratories). After washing, tissue was mounted onto slides, dehydrated in an ascending series of ethanol washes, coverslipped and imaged under brightfield microscopy on a Zeiss Axioskop.

c-fos immunoreactivity (FOS-IR) in saline versus SB-399885 treated rats was surveyed and mapped across the brain to identify sites through which 5-HT_6_R may mediate its appetitive effects. Since consistent SB-399885-related changes in FOS-IR were obtained in rats treated at the onset of the light and dark cycle, only data related to rats treated at the onset of the light cycle are presented. Of particular interest was a significant increase in FOS-IR noted in brain regions associated with appetite such as the hypothalamus and brainstem (Fig. 2). We next quantitatively assessed FOS-IR in feeding-related brain nuclei, the PVH, ARC, VMN, dorsomedial hypothalamus (DMH), dorsal raphe nucleus (DRN) and nucleus of the solitary tract (NTS) at three neuroanatomical levels as defined by the Paxinos and Watson rat brain atlas [17]. Specifically, we counted FOS-IR neurons at the following bregma levels: PVN, −1.72 mm, −1.80 mm, −1.92 mm; ARC, −2.40 mm, −2.92 mm, −3.84 mm; DMH, −3.00 mm, −3.12 mm, −3.36 mm; VMN, −2.28 mm, −2.64 mm, −3.00 mm; DRN −6.96 mm, −8.04 mm, −8.40 mm; NTS −12.60 mm, −13.80 mm, −14.04 mm.

A hypophagic dose of SB-399885 significantly increased FOS-IR in the PVH, in particular at −1.72 (*t*_10_ = 3.77, *p* = 0.01) and −1.80 (*t* test, *t*_10_ = 7.06, *p* < 0.0001) from bregma in rats treated at the onset of the light cycle (Fig. 3). Likewise, rats treated with 2 mg SB-399885 at the onset of the dark cycle also showed a significant increase in PVH FOS-IR at −1.80 mm (*t*_4_ = 3.06, *p* = 0.02) compared to saline. The PVH is well established as a critical regulator of anorectic behaviour. Specifically, PVH lesion studies, PVH morphological genetic disruption via *Sim1* and injection of anorectic factors into the PVH all support a primary role of this nucleus in energy balance [18,19]. Furthermore, restoration of melanocortin4 receptors (MC4Rs) in the PVH of MC4R null mice is sufficient to reinstate d-fenfluramine anorexia, demonstrating the importance of the PVH in 5-HT hypophagia [20].

In contrast to the PVH, SB-399885 produced no observable changes in FOS-IR within the canonical satiety centre of the hypothalamus, the ARC (Fig. 3; [1]), where 5-HT_6_R are expressed [12]. The ARC-PVH circuit is a key component of 5-HT, 5-HT_2C_R and 5-HT_1B_R agonist hypophagia [6,7,20,21]. Specifically, 5-HT via 5-HT_2C_Rs activates anorectic ARC pro-opiomelanocortin (POMC) neurons projecting to the PVH and via 5-HT_1B_Rs inhibits hunger promoting ARC agouti-related peptide (AgRP)/GABA neurons. A lack of increased FOS-IR in the ARC following 5-HT_6_R antagonist administration does not preclude action within this nucleus. Like 5-HT_1B_R agonists, 5-HT_6_R antagonists may prevent the activity of hunger stimulating ARC AgRP/GABA neurons projecting to the PVH. This is consistent with the notion that 5-HT_6_R antagonists reduce food intake by influencing GABAergic neurotransmission, as 5-HT_6_Rs are expressed on GABA neurons in other brain regions [9] and pre-treatment with 5-HT_6_R antagonist Ro 04-6790 attenuates GABA_A_ receptor agonist muscimol-induced hyperphagia [22]. In this context, our observation that 5-HT_6_R antagonist administration increases PVH neuronal activation may be via blockade of 5-HT action on inhibitory ARC AgRP/GABA neurons, promoting the disinhibition of downstream anorectic PVH neurons. This is a possible mechanism through which 5-HT_6_R-antagonist hypophagia may be achieved.

SB-399885 did not significantly influence FOS-IR in other hypothalamic energy balance associated regions, the VMN or DMH (Fig. 3). Nor did it influence FOS-IR in the DRN, a key site where 5-HT is synthesised that projects to the hypothalamus (including the PVH) and NTS (Fig. 3). In contrast, SB-399885 did substantially increase FOS-IR in the brainstem NTS compared to vehicle treatment in rats treated at the onset of the light and dark cycle. Specifically, 2 mg/kg SB-399885 increased neuronal activation by 3-fold in the NTS at −13.80 mm from bregma (*t*_10_ = 5.45, *p* = 0.001), and to a lesser extent, the caudal portion at −14.04 mm (*t*_10_ = 3.10, *p* = 0.01) in rats treated at the onset of the light cycle (Fig. 3). Likewise, compared to saline, SB-399885 increased FOS-IR 2-fold at −13.80 mm from bregma (data not significant) and 3-fold at −14.04 mm (*t*_4_ = 2.493, *p* = 0.03) in rats treated at the onset of the dark cycle. The NTS represents an integrative node through which vagal, hormonal and chemical inputs converge to modulate feeding behaviour, with the preponderance of appetitive neurons located at the level of the area postrema (AP). These include energy status signals such as 5-HT, leptin, ghrelin, POMC, cholescytokinin (CCK) and glucagon-like peptide (GLP-1) that are expressed and/or acting within the NTS [23]. Antagonism of 5-HT_6_R signalling therefore may act to suppress inhibitory input onto satiety-related NTS neurons, thus promoting their activity and anorectic influence. Equally, PVH processing has been reported to influence appetitive signalling in the NTS [24] suggesting that SB-399885-mediated activation of PVH neurons may also influence the activity of satiety-related neurons in the NTS and vice versa through a PVH-NTS reciprocal circuit.

Pharmacological compounds modulating endogenous 5-HT bioavailability such as d-fenfluramine and sibutramine were used for the treatment of human obesity, but were withdrawn from clinical use due to off-target effects. A key component of the therapeutic effect of these compounds is mediated via activation of 5-HT_2C_Rs. Capitalising on this therapeutic mechanism, Arena's 5-HT_2C_R agonist lorcaserin (Belviq) was launched in the USA in the summer of 2013 for obesity treatment. However, 5-HT_6_R antagonists have also been demonstrated to reduce food intake and body weight gain in comparable proportions to 5-HT_2C_R agonists in preclinical studies [9,10,22,25] and represent a further opportunity for obesity treatment drug development. However, the physiological mechanism through which 5-HT_6_R antagonists reduce food intake, achieved via advanced satiety, reduced hunger, induction of nausea, reduced hedonic properties, or induction of behaviours that interfere with feeding (e.g. sedation) remains to be determined. These findings will impact the utility of 5-HT_6_R antagonists for obesity treatment.

Here we demonstrate that 5-HT_6_R antagonist hypophagia shares the activation of two common brain regions with 5-HT_2C_R agonists, the PVH and NTS [16,20], brain regions which are critical for the homeostatic regulation of appetite. These data suggest a potential mechanistic convergence of 5-HT_2C_Rs and 5-HT_6_Rs in critical energy balance nodes, the PVH and NTS.

In conclusion, we report that the selective 5-HT_6_R antagonist, SB-399885, produces a dose-dependent decrease in food intake in rats which is associated with a significant increase in neuron activity in the PVH and NTS. The findings presented here provide insight into the neural circuitry engaged by 5-HT_6_R pharmacological blockade and highlight the PVH and NTS as potential mediators of the coordination of 5-HT_6_R antagonist-induced appetite suppression.

## Figures and Tables

**Fig. 1:**
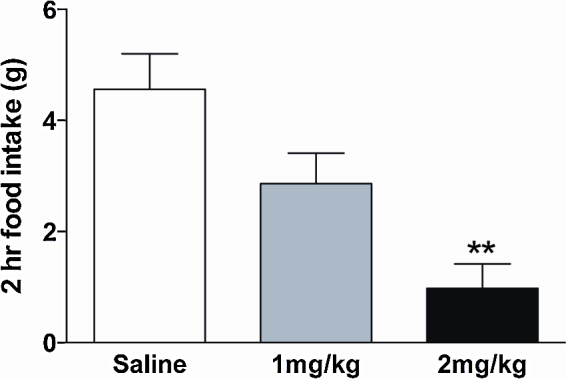
SB-399885 dose-dependently reduced food intake. 0.9% saline (white bar) or SB-399885 at doses of 1 (light grey bar) or 2 (black bar) mg/kg, i.p. was administered at the onset of the dark cycle and food intake measured over the next 2 h in rats. Data are presented as mean ± S.E.M. Statistics, one way ANOVA, *F*_2,18_ = 9.35, *p* = 0.016. **p < 0.01 compared to saline treatment.

**Fig. 2:**
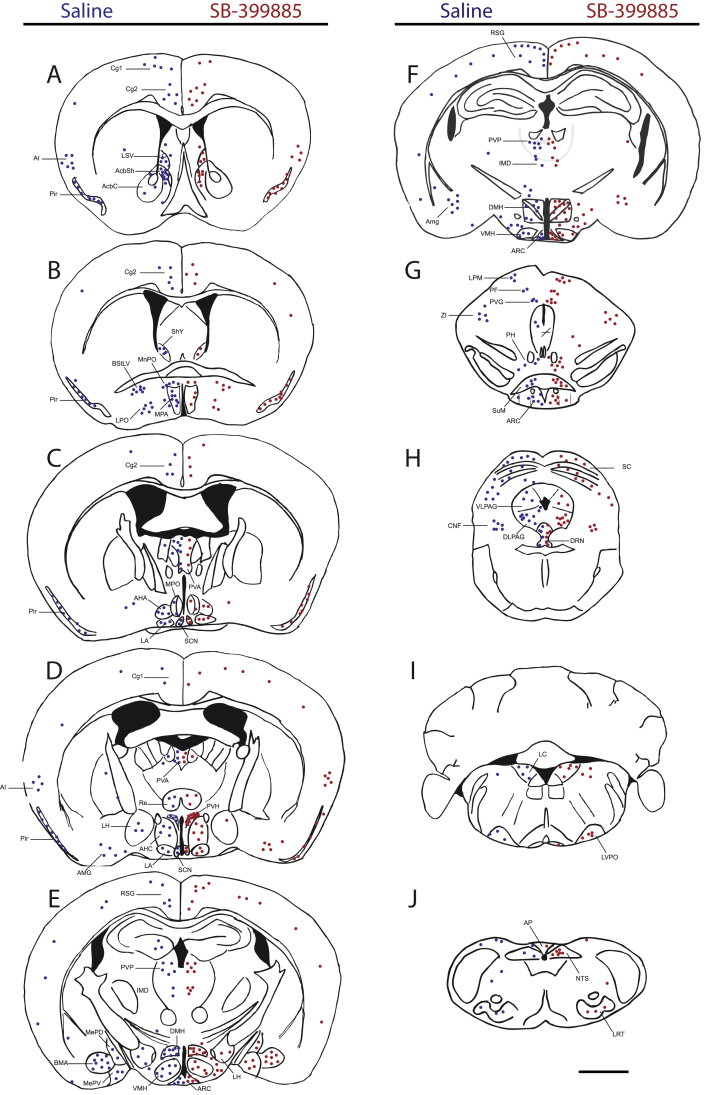
A series of photomicrographs comparing whole-brain FOS-IR in response to saline (blue dots on left, *n* = 3) versus 2 mg/kg SB-399885 (red dots on right, *n* = 3), i.p. Brain sections are arranged rostral to caudal (A–J). One blue or red dot indicates five FOS-IR cells. Scale bar = 1 mm. *Abbreviations*: AcbSh, shell portion of the accumbens nucleus; AHA, anterior hypothalamic area, anterior part; AHC, anterior hypothalamic area, central part; AI, agranular insular coretx; AMG, amgydala; AP, area postrema; BMA, basomedial amygdaloid nucleus; BSTLV, bed nucleus of the stria terminalis lateral division ventral part; Cg1, cingulate cortex area 1; Cg2, cingulate cortex area 2; CnF, cuneiform nucleus; DLPAG, dorsolateral periaqueductal grey; IMD, intermediodorsal thalamic nucleus; LA, lateroanterior hypothalamic nucleus; LC, locus coeruleus; LPLC, lateral posterior thalamic nucleus; LPM, lateral posterior thalamic nucleus; LPO, lateral preoptic area; LRt, lateral reticular nucleus; LVPO, lateroventral periolivary nucleus; MePD, medial amygdaloid nucleus, posterodorsal part; MePV, medial amygdaloid nucleus; MnPO, median preoptic nucleus; MPA, medial preoptic area; MPO, medial preoptic nucleus; PVA, paraventricular thalamic nucleus, anterior part; PVG, periventricular grey; PF, parafascicular thalamic nucleus; PH, posterior hypothalamic area; Pir, piriform cortex; PVA, paraventricular thalamic nucleus, anterior; PVG, periventricular grey; PVP, paraventricular thalamic nucleus, posterior; Re, reuniens thalamic nucleus; RSG, retrosplenial granular cortex; SC, superior colliculus; SCN, suprachiasmatic nucleus; SHy, septohypothalamic nucleus 27; SuM, supramammillary nucleus; VLPAG, ventrolateral periaqueductal grey; ZI, zona incerta.

**Fig. 3:**
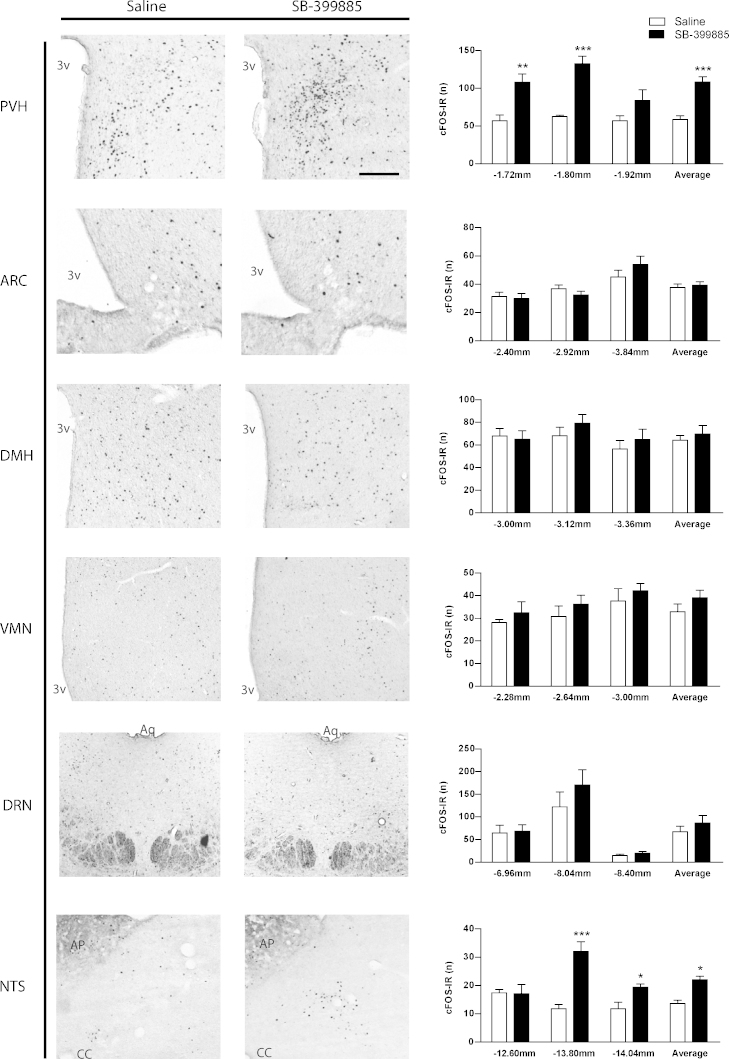
SB-399885 significantly increased FOS-IR in the PVH and NTS compared to saline. Counts of FOS-IR (*n*) in the PVH, ARC, DMH, VMN, DRN, and NTS following treatment with saline (white bar) or 2 mg/kg SB-399885, and accompanying representative photomicrographs. SB-399885 significantly increased FOS-IR in the PVH at −1.72 mm (*t*_10_ = 3.77, *p* = 0.01) and −1.80 mm (*t* test, *t*_10_ = 7.06, *p* < 0.0001) from bregma, and at the level of the area postrema in the NTS at −13.80 mm (*t*_10_ = 5.45, *p* = 0.001) and −14.04 mm (*t* test, *t*_10_ = 3.1, *p* = 0.01). Scale bar = 50 μm. Data are presented as mean ± S.E.M., *p < 0.05; **p < 0.01; ***p < 0.001 compared to saline treatment. *Abbreviations*: PVH, paraventricular nucleus of the hypothalamus; ARC, arcuate nucleus of the hypothalamus; DMH, dorsomedial nucleus of the hypothalamus; VMN, ventromedial nucleus of the hypothalamus; DRN, dorsal raphe nucleus; NTS, nucleus of the solitary tract; cc, central canal; Aq, aqueduct and AP, area postrema.
